# Odor-Sensing System to Support Social Participation of People Suffering from Incontinence

**DOI:** 10.3390/s17010058

**Published:** 2016-12-29

**Authors:** Alvaro Ortiz Pérez, Vera Kallfaß-de Frenes, Alexander Filbert, Janosch Kneer, Benedikt Bierer, Pirmin Held, Philipp Klein, Jürgen Wöllenstein, Dirk Benyoucef, Sigrid Kallfaß, Ulrich Mescheder, Stefan Palzer

**Affiliations:** 1 Laboratory for Gas Sensors, Department of Microsystems Engineering, University of Freiburg, Freiburg 79110, Germany; alvaro.ortiz.perez@imtek.uni-freiburg.de (A.O.P.); kneer.j@gmail.com (J.K.); benedikt.bierer@imtek.uni-freiburg.de (B.B.); Juergen.Woellenstein@ipm.fraunhofer.de (J.W.); 2 Steinbeis Transfer Center, Social Planning, Qualification and Innovation, Meersburg 88709, Germany; Vera.Kallfass-deFrenes@stz-sozialplanung.de (V.K.-d.F.); info@stz-sozialplanung.de (S.K.); 3 Institute for Microsystems Technology, Mechanical and Medical Engineering, Furtwangen University, Furtwangen 78120, Germany; fil@hs-furtwangen.de (A.F.); mes@hs-furtwangen.de (U.M.); 4 Signal Processing Research Group (ReSP), Mechanical and Medical Engineering, Furtwangen University, Furtwangen 78120, Germany; pirmin.held@hs-furtwangen.de (P.H.); mail@ph-klein.de (P.K.); bed@hs-furtwangen.de (D.B.); 5 Fraunhofer Institute for Physical Measurement Techniques (IPM), Freiburg 79110, Germany

**Keywords:** ambient assisted living, inkjet-printed p-type semiconducting metal oxide, hotplate, autonomous odor detection device, wireless connectivity

## Abstract

This manuscript describes the design considerations, implementation, and laboratory validation of an odor sensing module whose purpose is to support people that suffer from incontinence. Because of the requirements expressed by the affected end-users the odor sensing unit is realized as a portable accessory that may be connected to any pre-existing smart device. We have opted for a low-cost, low-power consuming metal oxide based gas detection approach to highlight the viability of developing an inexpensive yet helpful odor recognition technology. The system consists of a hotplate employing, inkjet-printed p-type semiconducting layers of copper(II) oxide, and chromium titanium oxide. Both functional layers are characterized with respect to their gas-sensitive behavior towards humidity, ammonia, methylmercaptan, and dimethylsulfide and we demonstrate detection limits in the parts-per-billion range for the two latter gases. Employing a temperature variation scheme that reads out the layer’s resistivity in a steady-state, we use each sensor chip as a virtual array. With this setup, we demonstrate the feasibility of detecting odors associated with incontinence.

## 1. Introduction

Physical disabilities and aging can cause the loss of control of the bladder and intestinal functions. Affected persons often cannot perceive the odors related to their own excretions. Besides, many are unable to verbalize explicitly the need of nursing care [1]. One the other hand, a delay in incontinence care involves physical health risks and may cause emotional and mental health issues of the persons concerned, which in turn also affects their relatives and also healthcare professionals. Besides, the sensing of excretions’ odor is also a critical issue for stoma patients. To improve these situations, odor detection technologies are a possibility and the objectives of an early detection and signalization of excretion odors by a sensor device include:

- Avoiding social insecurity and shame;
- Unconfined participation in social life;
- Appropriate or improved care;
- Easing the burden of caregivers.

**Figure 1:**
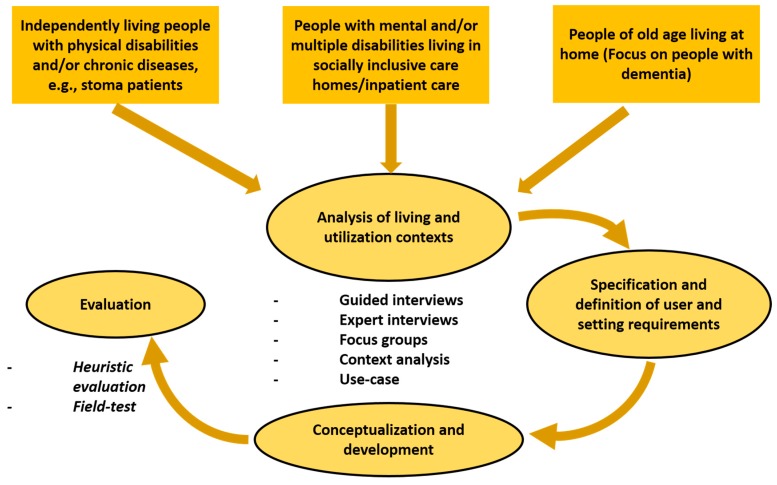
Visualization of the iterative user-centered research design that has been adopted within this research and development effort.

However, it is fundamental that advances in technological capabilities are tailor-made to the needs of end-users to ensure acceptance of innovations. As a result, an iterative research process has been adapted during development of the odor sensing system to prevent rejection of possible technology-based solutions by the affected person. For the results presented here, we have adopted a setting- and user-centered approach, which includes the early and continuous involvement of affected people, considering their living and care environment during the process, as well as the empirical examination and evaluation. These key principles are at the heart of the research and development efforts presented here. Figure 1 visualizes our approach which has involved an inter-and transdisciplinary team of experts and laymen.

The needs of potential groups of users was analyzed by applying established research methods [2,3,4,5,6] such as guided interviews, focus groups, expert interviews, context analysis, and use-cases. The analysis of living and utilization contexts showed that significant gains in terms of quality of life may be achieved with a miniaturized, mobile odor sensing device for people with mental and multiple disabilities that live in socially inclusive care homes/inpatient care. The specification and definition of user and setting requirements led to interesting and valuable results, such as findings about stoma patients. Excretions in stoma pouches often lead to a strong odor formation. Since the human olfactory system is strongly adaptable, stoma patients become unable to perceive surrounding odors over time. Due to this adaption of odor connected with uncertainty about their own excretions, stoma patients often isolate themselves or even suffer from social exclusion. Here, a mobile odor sensor can act as a neutral notification system providing the opportunity to restore emotional security. 

In the past and currently, extensive research and development efforts have been and are aimed at developing so-called electronic noses [7]. Main application areas include health, security, and safety scenarios [8] and often focus on the highly selective detection of a few, well-defined gaseous compounds [9,10,11]. However, because of the complexity of situations in the real world, data analysis using electronic noses to identify a situation might prove advantageous as compared to analyzing specific gas concentrations [12]. Also, the mimicking of the human olfactory sense is being worked on, even though it is inherently difficult for an impartial machine to generate the highly individual perception of a smell [13]. The methods employed to achieve the specific goals of a given application include infrared, catalytic, electrochemical, mass sensing, or using semiconducting metal oxides [14,15]. Metal oxide-based detectors are particularly appealing, because of their long life time, good sensitivity and low cost production [16]. In order to increase the selectivity of metal oxide based sensors and utilizing the temperature dependence of surface reactions [17,18], the use and optimization of temperature modulation techniques has been investigated for several decades now and continues to be an active research field [19,20]. As compared to using spatial temperature gradients [21] on a sensor chip, the variation of temperature on a hotplate allows for reducing the overall power consumption using a quasi-static operation for each temperature value.

In our contribution, we do not aim for emulating the human olfactory sense, whose working principle relies on the binding of odorant molecules to highly specific receptors in the human nose [22]. Instead, the goal here is to reliably detect and discriminate three situations, namely the occurrence of smells associated with urine, feces and any another situation. This approach is especially advantageous in environments where typically large amount of cleaning agents are being used. To this end it is important to first analyze the constituents of the respective odors in order to be able to choose suitable detection techniques. Table 1 gives an overview about the main gases occurring in both, urine and feces, as well as odors.

From this list it becomes apparent that sulfidic molecules and amines play a central role for the odor perceived by the human olfactory system in the relevant scenarios. Furthermore, the gas composition for situations where an odor linked to urine or feces occurs differ from odors, which in turn should make it possible to reliably detect the associated situations. 

The interviews of different user groups yielded as the most important issue for the users the need to inconspicuously provide information about occurrence of incontinence related odors directly to the user and if desired also to a supporting person (e.g., caretaker). Therefore, the sensor system is supposed to give discrete notification to mobile, active, and socially participating people who use it whenever other people might perceive an odor related to urine or feces. The novel system has been designed according to the end-user needs and in close collaboration with them.

**Figure 2:**
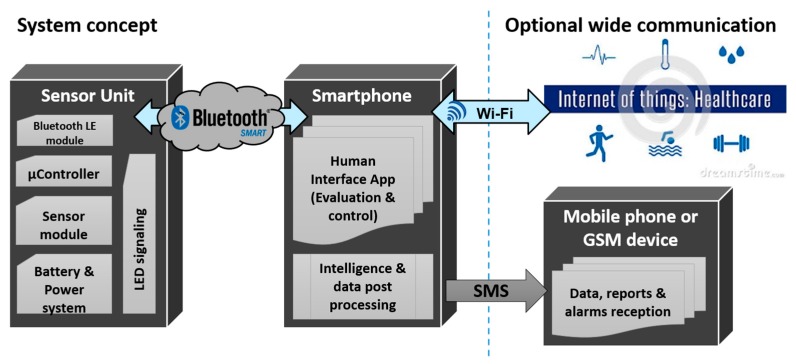
The concept for the technical solution is based on an add-on device to already available infrastructure, in this case a smartphone. Using Bluetooth LE protocol the raw data from the sensing components are transferred to the smart device, where the powerful CPU is used to perform data analysis tasks. All user-notification channels are controlled by the smart device.

To achieve this, we have established a concept that relies on using any pre-existing smart device of the user and an odor detection unit as accessory for the smart device. The concept of the overall system is depicted in Figure 2. 

On the one hand, this approach allows the use of the smart device’s large computing power as well as all the human-machine interfaces state-of-the-art mobile devices offer, including apps, messenger services, and acoustic and tactile signaling. On the other hand, the sensor device might be fastened at arbitrary locations and independent of the smart device (e.g., a smart phone) within the communication range of the wireless protocol used.

The gas detection is based on semiconducting metal oxide technology, which allows for low-cost production, miniaturization, and portable application. The gas sensitive layers are each deposited using an inkjet printing process allowing for deposition of the functional materials onto arbitrary sensing structures. While previous reports on the gas sensitive behavior towards malodorous volatile organic compounds focus mostly on n-type semiconducting metal oxide layers, e.g., using SnO_2_ [31] or ZnO [32], or mixtures of metal oxides such as SnO_2_ and CuO [33] we use the respective gas sensing properties of ink-jet printed, p-type semiconducting CuO and CTO because of their weaker sensitivity towards humidity. We do this to be able to investigate whether the anticipated enhancement in robustness in future real-world deployments is worth the cost of lower sensitivity. To our best knowledge, the gas sensitive behavior of both gas sensitive materials towards methylmercaptan and dimethylsulfide has not been reported before. The experimental results provide insights in terms of the influence of humidity. Using a temperature variation protocol, we convert each sensing layer into a virtual sensor array and show that the additional information obtained this way increases the selectivity of the layers. 

The good baseline stability of the metal oxide devices should allow for producing reproducible and robust real-world devices. As a result, we can demonstrate an end-used driven design of an autonomous, stable incontinence sensor module based on metal oxide technology for the first time.

## 2. Materials and Methods 

**Figure 3:**
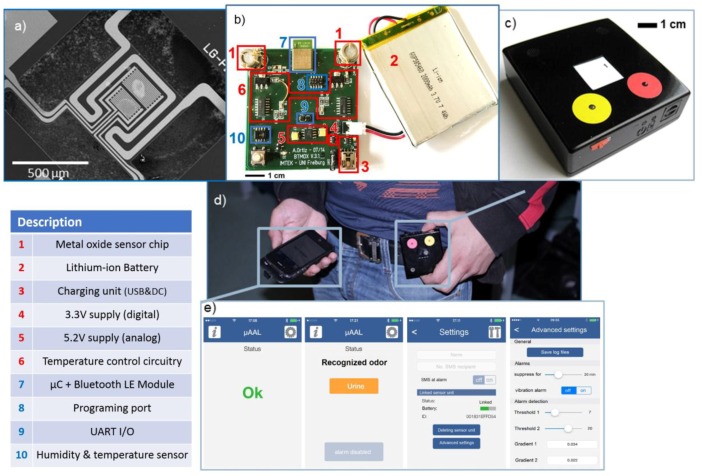
(**a**) The micro-machined suspended hotplate sensor chip used in the setup features a heating structure and two interdigitated electrode structures. The image has been taken using a scanning electron microscope at 80× magnification; (**b**) The complete hardware of the sensor module (PCB & battery); (**c**) Sensor module package with LEDs to indicate incontinence related odors to user directly; (**d**) Photo of the system as it may be used with a commercially available smartphone and the sensor module; (**e**) Example of a possible realization of the human interface.

The odor sensing unit is built around two ink-jet printed, low-power consuming micro-machined hotplate chips that each employ a p-type semiconducting metal oxide as gas sensitive layer. The low-power consuming devices consist of a platinum heater and two interdigitated electrode structures as shown in Figure 3a, which provide an interface to read out the resistivity of the gas-sensitive metal oxide layers. Details on the fabrication and characterization of the micro-electro-mechanical system (MEMS) devices are presented in [34]. The MEMS chips are mounted in a 12-pin TO5 housing and connected to it via ball bonding. Because of the high thermal insulation of the hotplate devices, neither the surrounding electronic components nor the chip mount itself are heated up. As gas sensitive elements we have used layers consisting of copper(II) oxide (CuO) [35] and chromium-titanium oxide (Cr_2−*x*_Ti_*x*_O_3+*z*_/CTO) [36]. We have chosen CuO because of its reaction towards sulfidic molecules [37] and CTO because of its relatively small response towards water [38]. The production of the inks follows our standard approach employing a dual step process as described in [39]: After generating a base dispersion using a wet grinding process employing a Retsch PM 100 planetary mill, polyethylene glycol (PEG) 400 and deionized water (DI-H_2_O) are added and mixed in order to match the viscosity specifications of the deployed Dimatix DMP2831 printer system [40]. Both CuO and CTO layers are printed in six stacked layers on top of the interdigitated Pt-electrodes of the MEMS chip. We determine the resistivity of the layer by means of a time-to-digital conversion method [41]. This approach minimizes the power consumption and the cost as compared to using analog-to-digital converters (ADC). Further information about the ambient air conditions is obtained by determining the ambient temperature and humidity using a commercially available digital sensor from Honeywell International, Inc (Morris Plains, NJ, USA) (HumidIcon^TM^ 6100 Series [42]) with an I2C bus. Control and read-out of all system components is achieved using a nRF51822 micro-controller (µC) from Nordic Semiconductor (Trondheim, Norway) [43]. This choice offers a convenient way to establish Bluetooth low energy (Bluetooth LE) connectivity since this feature is integrated in the chip. A 32-bit ARM R Cortex^TM^ (Cambridge, UK) M0 CPU with 256 kB Flash +16 kB RAM provides enough capabilities to readily perform all basic tasks, namely temperature control of the metal oxide sensor chip, read-out of the resistivity, the digital humidity and temperature sensor. The overall power supply of the odor sensing module is achieved with a lithium-ion polymer battery from Unionfortune Electronic Co., Ltd (Guangdong, China) [44] with a total charge of 2000 mAh at 3.7 V output voltage. Re-charging is possible via a mini USB port. The electronics and all sensing components are packaged in a (7 × 6 × 2.5) cm³ housing made using the MultiJet Modeling (MJM) technique and using VisiJet EX200 as plastic material [45] (Figure 3b). The MJM-technique allows 3-D prototyping of complex structures with high resolution of down to 30 µm, which is needed to package the different components of the system including gas inlet channels and interfaces. To provide a fully-opaque, black package and a chemically resistant surface that does not influence the gas sensing chips, the plastic housing was coated with a two component acrylic lacquer. The acrylic lacquer coating reduces the outgassing of the plastic material which is a serious disadvantage when using materials suitable for rapid prototyping. Using a Bluetooth LE connection, the data is transferred between the odor sensing module and the smart device, which in this case is an iPhone4. We have implemented the possibility to send the result of the raw data evaluation back to the sensor module, where it triggers signaling light-emitting diodes (LEDs) indicating an alarm as well as a stable connection. While this has no impact on the performance of the system and is unnecessary from a technical point of view, it was included because users wish to assert themselves that the odor module is working properly and to get an indication directly on the device if incontinence-relevant odors are sensed or not. The main human interface, however, is an application installed on the smart device. A screenshot of a possible realization is displayed in Figure 3e. The app has been programmed to highlight the possibilities of realizing an interface with the user and is divided into different screens to show the current odor status, to change the detection threshold values, and to activate further notification channels such as a vibrational alarm, respectively. The complete sensing system is shown in Figure 3 and the sensing module is realized in a single printed circuit board (PCB). Analysis of the sensor module’s data is performed on the connected device and the result is displayed in a custom built app that may be individualized. 

### 2.1. Operational Protocol

Metal oxide-based gas sensors are known to have poor selectivity but are highly sensitive to a large number of oxidizing and reducing gases. Also, because the adsorption processes at the surface are temperature dependent, a single metal oxide layer reacts differently at different temperatures. By employing a specific temperature protocol in which quasi steady state conditions over a large range of temperature range are achieved we convert each chip into a virtual array while at the same time reducing the overall power consumption. The MEMS platform allows for fast temperature variation and here we implement a scheme where we set the temperature of the two chips to four temperature levels between 150 °C and 420 °C. The results presented here only make use of one gas sensitive layer (CuO or CTO) per chip. For this reason, the chip employing CuO as a gas sensitive layer is denoted CuO chip, and the other one is denoted CTO chip.

The temperature of each hotplate is set via a voltage-controlled current source (VCCS), built with a simple op-amp and a load resistor, whose control signal is generated through a filtered pulse width modulation (PWM) signal generated by the µC. To determine the temperature of the hotplate, the resistivity of the heating structure is determined by the known applied current, which is set by the VCCS, and the heater voltage determined by the µC through an external op-amp in differential configuration and the internal ADC of the µC. Because an active control of the temperature will reduce the data acquisition frequency and increment the power consumption using this configuration, we have divided the operational protocol into two parts. After switching the device on, it is calibrated by creating a look-up table. For this, several PWM signals are generated and the heater’s resistivity is determined, which corresponds to a well-defined sensor temperature. The sensor system then automatically enters the measurement mode, where the PWM signal is generated according to the look-up table. Within every measurement cycle, both sensor chips are operated at four different temperatures each—i.e., 150 °C, 225 °C, 260 °C and 330 °C for the CuO chip and 260 °C, 330 °C, 360 °C and 420 °C for CTO, respectively. Each temperature step is held for 300 ms before triggering the time-to-digital read-out of the resistivity of the gas sensitive layer. This results in a variable time for each temperature step depending on the layer’s resistivity. The total time *t*_TTD_ necessary for resistivity determination (using a 1 nF capacitor) varies between 2.9 ms and 200 ms corresponding to 10 kΩ and 10 MΩ, respectively. The resistivity value used for analysis is then calculated from an average of 16 repetitions of the time-to-digital measurement, which results in a duration of 3.2 s for a resistivity value at most. Afterwards, the dummy resistance is determined using the same method. Each time a measurement is triggered, the sensor temperature is checked to ensure it is at the desired value, according to the values and parameters defined by the temperature control unit calibration module. If this test fails, the value is discarded. The calibration function is triggered when the ambient temperature sensor detects drastic changes in the ambient conditions exceeding 10 °C or if measurement is discarded twice in a row. 

**Figure 4:**
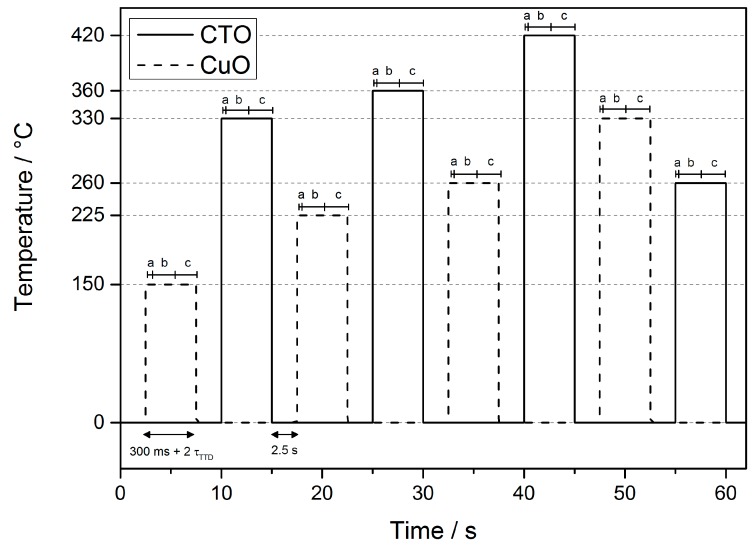
Temperature protocol used in normal operational mode. Each temperature step comprises 300 ms preheating time (a), and two (b, c) time to digital measurement phases of both sensor resistive layers. A 2.5 s time gap between every step is used for wireless data transmission. Please note that the times b and c depend on the layer’s resistivity values and consequently vary considerably. As a consequence, the total duration time of the measurement cycle is not constant.

Once the resistivity values of one chip have been acquired, the hotplate is turned off, the data are transferred to the smart device, and the second chip is utilized. Figure 4 highlights the protocol as we use it in the operational mode. Because the time-to-digital method is inherently restricted in its acquisition speed by the probed resistivity and the fixed values of the circuitry, we are unable to record transient responses of the resistive layers upon temperature change. Therefore, we only use steady-state values of the resistivity, which we check to be a valid assumption for our system. While this scheme prevents us from using the transient response of the sensing layer upon temperature modulation, which would yield more features for pattern recognition [46], we still increase the information extracted by operating these virtual arrays at different temperatures [47]. 

**Figure 5:**
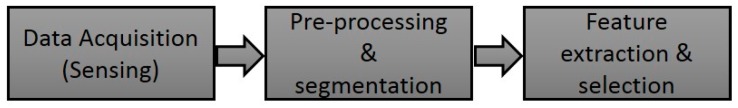
Block diagram of the data pre-processing used.

To evaluate the performance of the systems in terms of its capability to discriminate between the different situations, we have implemented a basic pattern recognition data processing [48,49]. The basic structure of the data processing is shown in Figure 5. After the data acquisition, a first pre-processing phase is carried out in order to filter data by removing outliers, which means that single data samples exceeding two times the standard deviation are disregarded. Then a feature extraction for dimensionality reduction aiming to find a suitable feature sub-space in order to be able to distinguish between patterns that belong to different classes is realized.

Because the sampling frequency of the time to digital method used is dependent on the actual resistivity value and the sensor system lacks a real-time clock (RTC), the readings from the eight virtual sensors need to be assigned a time. This is done by the smart device connected to the sensing module by assigning a time stamp to each sample as they are received, thus generating a unique time vector common for all of the virtual sensor´s data vectors as well as for the data vectors from the temperature and humidity sensor. After that, all vector values are equally weighted and a normalization is carried out, making all individual sensor values to range between [0, 1]:

(1) $$y_{S}^{k}=\frac{x_{S}^{k}-{min}_{\forall k}\left[x_{S}^{k}\right]}{{max}_{\forall k}\left[x_{S}^{k}\right]-{min}_{\forall k}\left[x_{S}^{k}\right]}$$

where $x_{S}^{k}$ is the kth sample of the response of virtual sensor ‘S’. After that, a linear transformation is used for visualization, analysis, and classification purposes as well as dimensionality reduction. In this case, a Fischer linear discriminant is used. This is also known as Linear Discriminant Analysis (LDA) technique and aims at finding a signal representation that establishes a transformation to maximize the inter-cluster distances between classes and minimizes intra-cluster distances within a given class [46].

### 2.2. Validation Experiments

To comply with user requirements, the battery-powered odor sensing component’s runtime without charging should at least match that of the mobile phone and, therefore, we have performed a power consumption test by recording the total current usage of a complete measurement cycle. 

We have also performed experiments to test our steady-state claim. To this end, we have produced another sensing chip using CuO and CTO as gas sensitive layer. We have performed an independent evaluation of the transient behavior of the gas sensitive layer upon temperature change using a Prema 5017 Digital Multimeter (PREMA Semiconductor GmbH, Mainz, Germany) and a data acquisition rate of eight samples per second. Each temperature step had a duration of 60 s and we have determined the minimum time necessary to reach steady-state values.

In order to test the robustness of our protocol, further investigate our steady-state approach, and check a stable operation, we have used the system to record a transient response towards exposure to varying levels of methylmercaptan from 1 ppm–5 ppm. The device performance of the prototype has been tested using well-defined gas mixtures produced by our custom-built apparatus to simulate real-world conditions in the lab [50]. Taking into account typical environmental conditions in real-world applications, we have opted for a characterization in two humidity regimes at 22% and 47% r.H., respectively. 

Next, we have tested further gases according to an analysis of the odorous components occurring in feces and urine. We measure the response to gas concentrations close to the head space concentrations detected in real samples [51,52,53]—i.e., 1 ppm for both methymercaptan and dimethlysulfide, and 20 ppm for ammonia to make sure the layers are capable of detecting these concentrations. 

In a final step, we have evaluated whether or not the information collected by the sensor module is able to distinguish the gas matrices associated with the target odors. We have exposed the system to atmospheres generated by pure samples of water, urine, and feces. Finally, we have collected data during the repeated exposure of the sensor module towards 10 min in laboratory air, above a water reservoir, a urine reservoir, and a feces reservoir.

## 3. Results

### 3.1. Operational Validation

**Figure 6:**
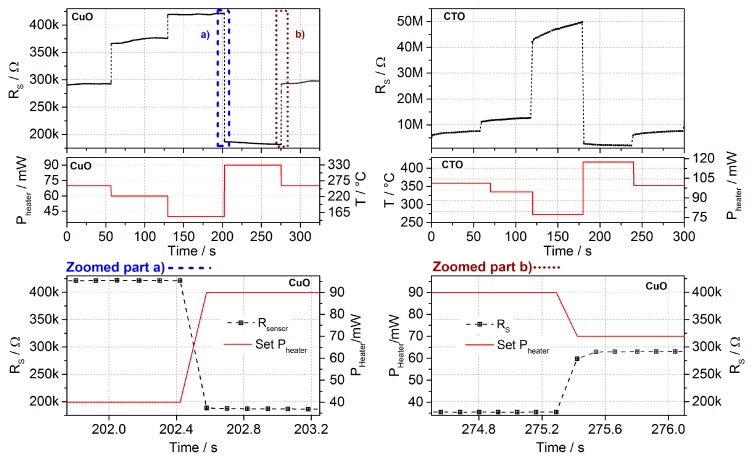
(**Top**) Evolution of the layer’s resistivity at different driving temperatures, which are associated with the heating power consumption of the chip. (**Bottom**) Part a) and b) show a more detailed view of the resistivity evolution after a temperature step. It demonstrates that the new steady-state is achieved in less than 250 ms, for both, temperature rises and drops.

The time-resolved evolution of the sensing layer’s resistivity for the different temperatures within a measurement cycle during the validation experiments is shown in Figure 6. 

Even though the baseline resistivity of CuO and CTO differ by more than one order of magnitude when compared to the layers of the prototype, the results may be applied to the respective layers used in the prototype device without loss of generality for both, CuO and CTO. As can be seen in Figure 6, bottom, the new thermodynamic equilibrium of the surface adsorbates is achieved after less than 250 ms, which is indicated by the constant resistivity at that point. Considering the normal system operation scheme where the sensor is preheated for not less than 300 ms before measuring the resistivity, this confirms that the virtual sensor-array approach with reaching steady-state conditions is valid. CTO shows a slow, slight drift behavior upon temperature change, which we attribute to diffusion processes. Despite the slight drift, for CTO the states itself are repeatable as can be clearly seen from the repeated measurement cycles, which use this protocol. The resistivity of the CuO layer shows a slight drift occurring after several 10 s of seconds, which is, however, non-repeatable. We attribute this to the inevitable slow changes in the sensing layer, which would occur in a complete steady-state operation as well and which could be corrected for [53]. 

Furthermore, the results obtained in determining the layer’s resistivity with the time-to-digital conversion are equivalent to that of more sophisticated equipment such as the Keithley 2700, which we use in our fully automated gas measurement apparatus [50]. As a result of these measurements, we can assume that the mobile sensor module is capable of correctly determining the resistivity of the CuO/CTO layers over a wide range of values with high accuracy.

**Figure 7:**
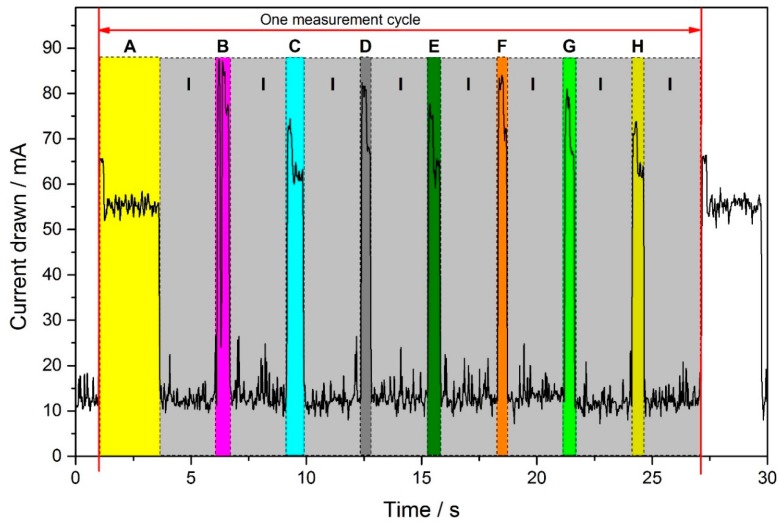
Current usage of a complete measurement cycle, compromising four different temperatures steps for each sensor, in which the resistivity of two sensitive layers from each sensor is estimated. The individual contributions are highlighted.

The total current consumption of the device during a complete measurement cycle is visualized in Figure 7. On average, the device consumes 113.85 mW, i.e., 30.77 mA at 3.7 V. We therefore estimate the total autonomy time of the odor sensing module according to Equations (2a) and (2b) to be 2.000 mAh/30.77 mA = 65 h, i.e., more than 2.5 days without recharging the battery:

(2a) Energy[Wh]=Capacity[Ah]·Voltage[V]

(2b) $$Battery\;Life\left[h\right]=\frac{BatteryCapacity\left[mAh\right]}{LoadCurrent\left[mA\right]}.$$

The total current consumption of the device exceeds that of the individual hotplate chips, which is due to the electronics design, which dissipates large parts of the excess current in other parts of the electronics. Because of the electronic circuitry, a considerable share of the total current consumption is lost in individual components of the electronics. Most notably, we have to employ an IC component to step-up the battery voltage due to the requirements of the sensor´s heater. 

### 3.2. Gas-Sensitive Characterization of the Metal Oxide Layers

**Figure 8:**
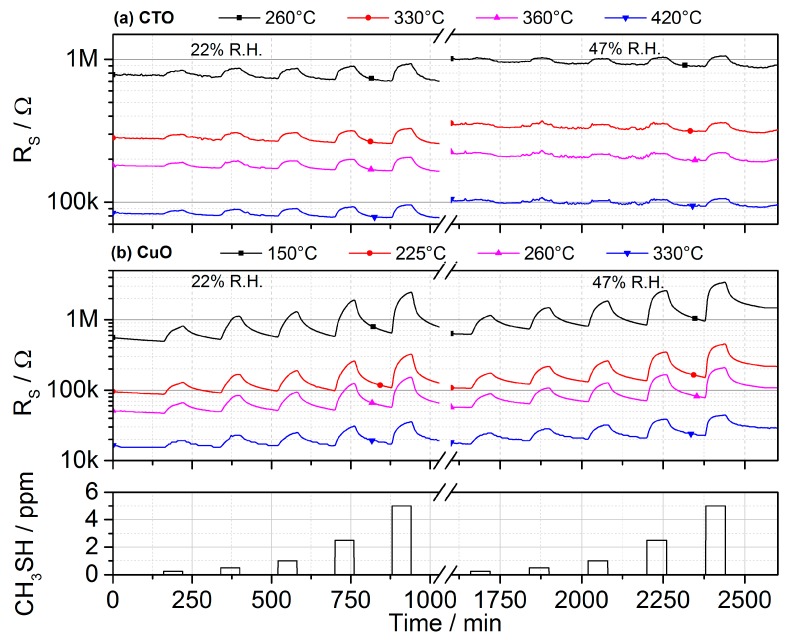
Recorded sensing resistance using the operational protocol for both metal oxide layers CTO (**a**) and CuO (**b**) for four temperatures, increasing CH_3_SH concentrations of 0.25, 0.5, 1, 2.5, and 5 ppm and two different humidity levels of 22% and 47% r.H. at 25 °C, respectively. The CuO layer shows poising effects upon exposure to methylmercaptan, especially at increased humidity levels, which is indicated by the non-returning baseline.

Figure 8 presents the gas sensitive reaction towards methylmercaptan for two humidity levels for both, CTO and CuO layers operated at the described temperature modulation protocol. This trace gas probably reacts with the oxygen species adsorbed on metal oxide surface and stepwise dehydrogenates. In [31,54] the net reaction is proposed to be:

(3) $$2\;{\text{CH}}_{3}\text{SH}+O_{2^{-}\left(\text{ads}\right)}\to {\text{CH}}_{3}{\text{SSCH}}_{3}+e^{-}+H_{2}O$$

Accordingly, the resistance of the p-type semiconducting metal oxides increases upon exposure towards CH_3_SH. 

**Figure 9:**
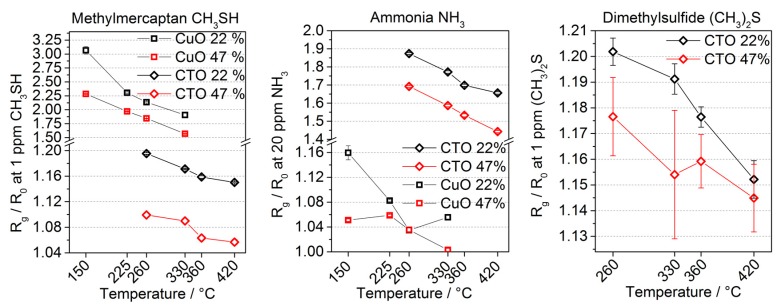
The gas sensitive reaction of the metal oxide layers towards test gas concentrations of 1 ppm of either dimethlysulfide or methylmercaptan and 20 ppm of ammonia. These concentrations are close to the head space concentrations reported in real samples, thus demonstrating detection limits are below these concentrations. The graphs also highlight the increase in selectivity achieved by using different temperatures to operate the metal oxide layers. On the right hand panel, again, no CuO reaction is shown, because the response was negligible.

The assessment of the capabilities of both layers to detect low levels of the most relevant trace gases with high reliability has been done by exposing the prototype system to head space concentrations reported in literature. The results also highlight the important influence of water vapor on the surface processes, which effects the layer’s response even though the influence is much weaker as compared to n-type semiconducting layers. We define the limit of detection towards a specific gas species as the concentration that causes a response R_G_/R_0_ exceeding 1.05, i.e., *R*_G_/*R*_0_ > 1.05, thus taking into account the baseline stability and noise of the resistivity reading. From the measurement data presented in Figure 9, we can assume a limit of detection of the system is well below headspace concentrations of real samples, i.e., one ppm for methylmercaptan and dimethylsulfide, and well below 20 ppm for ammonia [27,51,52]. While these performance data are above the recognition threshold of the human nose, they are in principle suitable to detect the different situations addressed here.

Analysis of gas sensitive performance also highlights the potential for an increase in selectivity by using different temperatures. The changes in sensor response as a function of the layer’s temperature are as high as 80% in our experiments and Figure 9 summarizes the sensor response for a fixed concentration of 1 ppm for the sulfidic molecules and 20 ppm for ammonia of each layer for the four different temperatures employed. So, the thermal modulation and steady-state read-out approach adopted here does allow for extracting additional information about the gas matrix and an increase in selectivity for distinguishing malodorous gas matrices.

### 3.3. Laboratory Validation with Real Odor Emitting Samples

**Figure 10:**
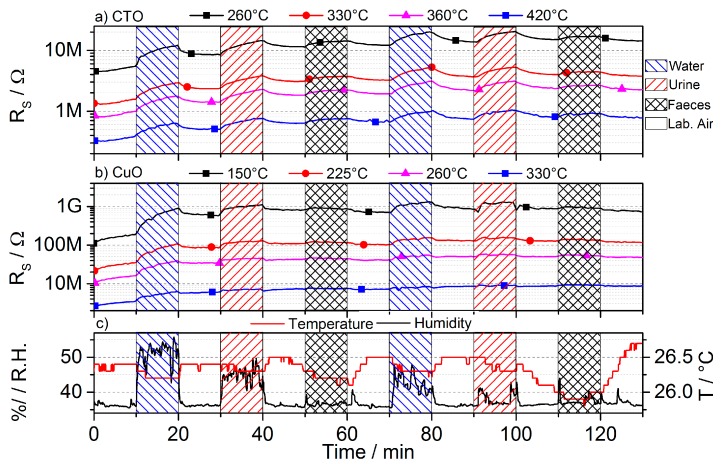
Sample sensor module raw data obtained after the exposure towards 10 min of humid laboratory air, above a urine reservoir, above a feces reservoir, and in laboratory air. (**a**) CTO and (**b**) CuO resistivity response (**c**) temperature and humidity.

The collected data during the repeated exposure of the sensor module towards laboratory air, above a water reservoir, a urine reservoir, and a feces reservoir is shown in Figure 10. The urine and feces samples have not been pre-treated or stored prior to the experiments but rather have been used as is with a waiting time of 15 min at most. 

**Figure 11:**
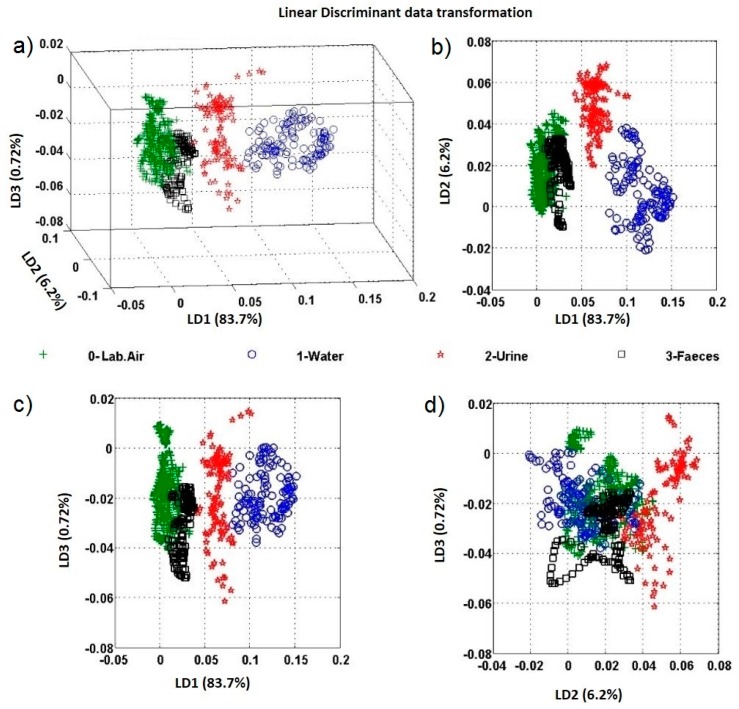
Linear discriminant analysis (LDA) of water, urine, and faeces probe measurement. (**a**) Shows the linear transformation of the raw data, which use the three largest discriminators (LD1, LD2, LD3), namely the eigenvectors with largest eigenvalues for the in-between-class and within-class scatter matrices. (**b**–**d**) show the LD1 vs. LD2, LD1 vs. LD 3, and LD2 vs. LD 3, respectively.

It shows, as expected, that humidity levels play a pivotal role in the resistivity of the metal oxide layers. The sensor data form the device have been treated according to the description in Section 2.1 and Figure 11 shows the result of projecting original data to the three largest discriminants, using data collected by all sensors (four virtual sensors per each sensor chip and the digital humidity sensor) on the device. It demonstrates that the four situations produce separate clusters. Still, the feces and laboratory air clusters are not well separated, which is probably due to the use of toilet paper with the feces and laboratory air samples as well as the generally lower volatility of components in non-liquid samples. However, this in turn means that the approach chosen here is essentially capable of detecting situations affecting end-users. 

## 4. Discussion

We have presented the design, development, and characterization of a portable, battery-powered odor sensing module that may be connected via Bluetooth low energy to any mobile device. Using low-power consuming MEMS devices with a metal oxide sensing layers placed on a hotplate, a runtime of more than two days has been achieved, which is about the utilization time of current smart devices without intermediate charging. The gas sensitive layers employed are capable of detecting relevant trace gases such as NH_3_, CH_3_SH, and (CH_3_)_2_S in relevant concentration ranges. An LDA shows that in near real-world situations the detection of target situations appears possible. Still, important issues need to be resolved prior to a field deployment. Currently, the detection limit is close to the target gas concentrations as encountered in real world samples. So, while the human odor threshold is still several orders of magnitude away, employing a device based on the technology presented here as an aiding device for stoma patients or elderly people suffering from incontinence seems possible. 

However, in order to successfully test the device in the field, we estimate that the sensor response needs to be improved by about one order of magnitude, which is feasible when using, e.g., catalysts. Additionally, the necessary detection limits will crucially depend on the location of the sensor in respect to the odor-producing source. To this end, one may place the device next to possible odor sources or employ pumping systems to provide the device with samples with high concentrations of the analyte. In this regard, we suggest that the mobile device will allow for a positioning close to the origin of the odor, thus providing relatively high target gas concentrations. Furthermore, the use of pre-concentrators is a possibility to increase the sensor signal and lower the detection limit. Adding highly selective elements, such as low-power consuming, room temperature electrochemical cells [55] or implementing novel methods for selective detection of hydrogen sulphide [39] may further increase the system’s capability to detect target situations.

The signal analysis may also have the potential to be considerably improved beyond using LDA and employing advanced, non-linear techniques such as multilayer perceptron (MLP). The employed algorithms will also have to be able to cope with the issue of changes in concentration associated with positioning of the device. 

Furthermore, the reproducible performance and calibration of various sensor modules will have to be investigated. We aim to deal with these issues based on the results presented in this paper. On top of that, non-technical issues such as user-acceptance, privacy concerns, and trust in new technologies in ambient assisted living settings will have to be investigated. Our current results constitute promising intermediate results aimed at addressing the issue of personal care and demonstrates the feasibility of employing low-cost, metal oxide-based sensing technology in a demanding measurement situation. 

## Figures and Tables

**Table 1:** Some of the most relevant odorous substances (odorants) occurring in human feces and urine and their odors [23,24,25,26,27]. The odor threshold values are taken from [28], except for benzaldehyde [29] and linalool [30].

Type of Substance	Perceived Fragrance	Chemical Formula	Natural Occurrence	Odor Threshold in Parts-Per-Million (ppm)
Methylmercaptan	Decayed cabbage	CH_3_SH	Urine/Feces	0.00007
Dimethylsulfide	Decayed vegetables	(CH_3_)_2_S	Urine/Feces	0.003
Dimethyldisulfide	n/a	C_2_H_6_S_2_	Urine/Feces	0.0022
Indole	Fecal/Flowery	C_8_H_6_NH	Odor/Feces	0.0003
Skatole	Fecal/Nauseating	C_9_H_9_N	Odor/Feces	0.0000056
Hydrogen sulfide	Rotten eggs	H_2_S	Urine/Feces	0.00041
Methylamine	Putrid/Fishy	CH_3_NH_2_	Feces	0.035
Ammonia	Sharp/Pungent	NH_3_	Urine/Feces	1.5
Acetaldehyde	Pungent/Fruity	CH_3_CHO	Feces	0.0015
Dimethylamine	Putrid/Fishy	(CH_3_)_2_NH	Odor/Feces	0.033
Triethylamine/Trimethylamine	Fishy/Ammonia	(C_2_H_5_)_3_N	Feces	0.0054
Benzaldehyde	Almond	C_7_H_6_O	Odor	0.00017
Limonene	Orange	C_10_H_16_	Odor	0.038
Linalool	Floral/Lavender	C_10_H_18_O	Odor	0.000047
